# Association of exposure to multiple perfluoroalkyl and polyfluoroalkyl substances and glucose metabolism in National Health and Nutrition Examination Survey 2017–2018

**DOI:** 10.3389/fpubh.2024.1370971

**Published:** 2024-04-03

**Authors:** Qinghua Tian, Yutong Yang, Qi An, Yang Li, Qingyao Wang, Ping Zhang, Yue Zhang, Yingying Zhang, Lina Mu, Lijian Lei

**Affiliations:** ^1^Department of Epidemiology, School of Public Health, Shanxi Medical University, Taiyuan, China; ^2^MOE Key Laboratory of Coal Environmental Pathogenicity and Prevention, Shanxi Medical University, Taiyuan, China; ^3^Department of Epidemiology and Environmental Health, School of Public Health and Health Professions, The State University of New York at Buffalo, Buffalo, NY, United States

**Keywords:** perfluoroalkyl and polyfluoroalkyl substances, National Health and Nutrition Examination Survey, glucose metabolism, least absolute shrinkage and selection operator, Bayesian kernel machine regression

## Abstract

**Objective:**

To investigate the relationships between perfluoroalkyl and polyfluoroalkyl substances (PFASs) exposure and glucose metabolism indices.

**Methods:**

Data from the National Health and Nutrition Examination Survey (NHANES) 2017–2018 waves were used. A total of 611 participants with information on serum PFASs (perfluorononanoic acid (PFNA); perfluorooctanoic acid (PFOA); perfluoroundecanoic acid (PFUA); perfluorohexane sulfonic acid (PFHxS); perfluorooctane sulfonates acid (PFOS); perfluorodecanoic acid (PFDeA)), glucose metabolism indices (fasting plasma glucose (FPG), homeostasis model assessment for insulin resistance (HOMA-IR) and insulin) as well as selected covariates were included. We used cluster analysis to categorize the participants into three exposure subgroups and compared glucose metabolism index levels between the subgroups. Least absolute shrinkage and selection operator (LASSO), multiple linear regression analysis and Bayesian kernel machine regression (BKMR) were used to assess the effects of single and mixed PFASs exposures and glucose metabolism.

**Results:**

The cluster analysis results revealed overlapping exposure types among people with higher PFASs exposure. As the level of PFAS exposure increased, FPG level showed an upward linear trend (*p* < 0.001), whereas insulin levels demonstrated a downward linear trend (*p* = 0.012). LASSO and multiple linear regression analysis showed that PFNA and FPG had a positive relationship (>50 years-old group: *β* = 0.059, *p* < 0.001). PFOA, PFUA, and PFHxS (≤50 years-old group: insulin *β* = −0.194, *p* < 0.001, HOMA-IR *β* = −0.132, *p* = 0.020) showed negative correlation with HOMA-IR/insulin. PFNA (>50 years-old group: insulin *β* = 0.191, *p* = 0.018, HOMA-IR *β* = 0.220, *p* = 0.013) showed positive correlation with HOMA-IR/insulin, which was essentially the same as results that obtained for the univariate exposure-response map in the BKMR model. Association of exposure to PFASs on glucose metabolism indices showed positive interactions between PFOS and PFHxS and negative interactions between PFOA and PFNA/PFOS/PFHxS.

**Conclusion:**

Our study provides evidence that positive and negative correlations between PFASs and FPG and HOMA-IR/insulin levels are observed, respectively. Combined effects and interactions between PFASs. Given the higher risk of glucose metabolism associated with elevated levels of PFAS, future studies are needed to explore the potential underlying mechanisms.

## 1 Introduction

Perfluoroalkyl and polyfluoroalkyl substances (PFASs) are a class of synthetic chemical that is widely used in various human production and daily life applications, such as paper, textiles, furniture, and foam fire extinguishers because of their thermal stability, hydrophobicity, and oil repellency (1–3). PFASs have high migration and contaminated ability and can be detected in environmental samples (such as water and soil), sera from various animal tissues, and human bodies (4–6). Additionally, PFASs have a significant bioaccumulation effect and a long half-life in the human body, making their degradation difficult (7). Animal experiments and epidemiological studies have demonstrated that PFASs have genotoxicity, reproductive toxicity, neurotoxicity, and developmental and endocrine-disrupting effects (8, 9).

There is growing evidence that PFASs are associated with a variety of health problems, with glucose metabolism disorder among them (10). Glucose metabolism disorder can cause many diseases, with diabetes being the most common, and has become a major public health issue (11). FPG, Insulin and homeostasis model assessment for insulin resistance (HOMA-IR) are important detection indices of glucose metabolism. FPG level was highly correlated with the presence of diabetic complications (12). Insulin is secreted by pancreatic β-cells, and human blood insulin levels can assess pancreatic β-cell function (13). Insulin resistance refers to the target organs of insulin action, such as liver, muscle and other reduced sensitivity to insulin action, and the normal physiological response of insulin cannot be performed (14). The most widely used assessment of insulin resistance is HOMA-IR (15). Early identification and control of these indices can reduce the harm of glucose metabolism disorder to the body and improve the prognosis.

Currently, epidemiological studies on the effects of PFASs on glucose metabolism have yielded conflicting and inconclusive results. The Diabetes Prevention Project analyzed the relationship between serum PFASs concentrations and blood glucose indices and found that perfluorooctane sulfonates acid (PFOS) and perfluorooctanoic acid (PFOA) concentrations were positively associated with the function of HOMA-IR, fasting blood glucose and β cells function (1). The level of serum PFASs 1871 adults was measured in the 2013–2014 National Health and Nutrition Examination Survey (NHANES) in the United States revealed that branched-chain PFOA level was positively correlated with increased FPG (10). However, a study on obese children in Ohio found no statistical significance between PFASs and blood glucose levels (16). Nelson et al. analyzed data from NHANES (2003–04) and found no significant association between the PFASs (PFOA, perfluorononanoic acid (PFNA), PFOS, and perfluorohexane sulfonic acid (PFHxS)) and HOMA-IR (2). Although several studies demonstrated a positive association between serum PFASs levels and glucose metabolism indices, various studies have also determined there to be a non-significant or inverse association. Therefore, further investigation into the relationships between PFASs exposure and glucose metabolism is warranted.

At present, the mechanism of PFASs affecting glucose metabolism is also not clear, and some researchers believe that it may be related to the activation of Peroxisome proliferator activated receptors (PPAR) (17, 18). PPAR belongs to the nuclear hormone receptor superfamily that regulates lipid, hormonal, and glucose metabolism and is considered possibly the major target of PFASs (19). PFASs can activate signaling pathways mediated by all PPAR isoforms (PPARα, PPARβ, PPARγ) (20). Moreover, PPARα may be a preferential target for PFAS above the other PPAR isoform (21). Toxicological studies have found that PFOA can also increase insulin sensitivity and glucose tolerance in mice by affecting the PI3K-AKT signaling pathway in the liver, causing an increase in fasting blood glucose level and a decrease in liver glycogen content in mice (22).

Additionally, most of the previous studies have focused on the biological toxicity of individual PFASs; however, in real-life settings, multiple PFASs often co-exist and interact during exposure, uptake, and metabolism processes, and this interaction can result in complex effects on body glucose metabolism. Currently, the specific effects of combined exposure to multiple PFASs on glucose metabolism remain unknown. Therefore, to provide new evidence on the relationships of PFASs exposure and glucose metabolism, we aimed to examine the relationships between exposure to multiple PFASs and glucose metabolism indices in this study by analyzing the NHANES data from 2017 to 2018, using the Least absolute shrinkage and selection operator (LASSO) and multiple linear regression analysis and Bayesian kernel machine regression (BKMR) models.

## 2 Materials and methods

### 2.1 Study population

Data on the study participants were obtained from the NHANES databases. NHANES is a unique 2 years cross-sectional survey of the health and nutrition status of the U.S. population that collects data on demographic, socioeconomic, and health-related issues through interviews, standardized exams, and biometric specimen collection. The health screening was conducted at a mobile Screening Center (MEC) after the participants had already participated in a household interview. The methods and processes used by NHANES for data collection are available on NHANES website^1^. In the current study, we used data from 2017–2018 wave which is the latest test data on PFASs in NHANES.

**Figure 1:**
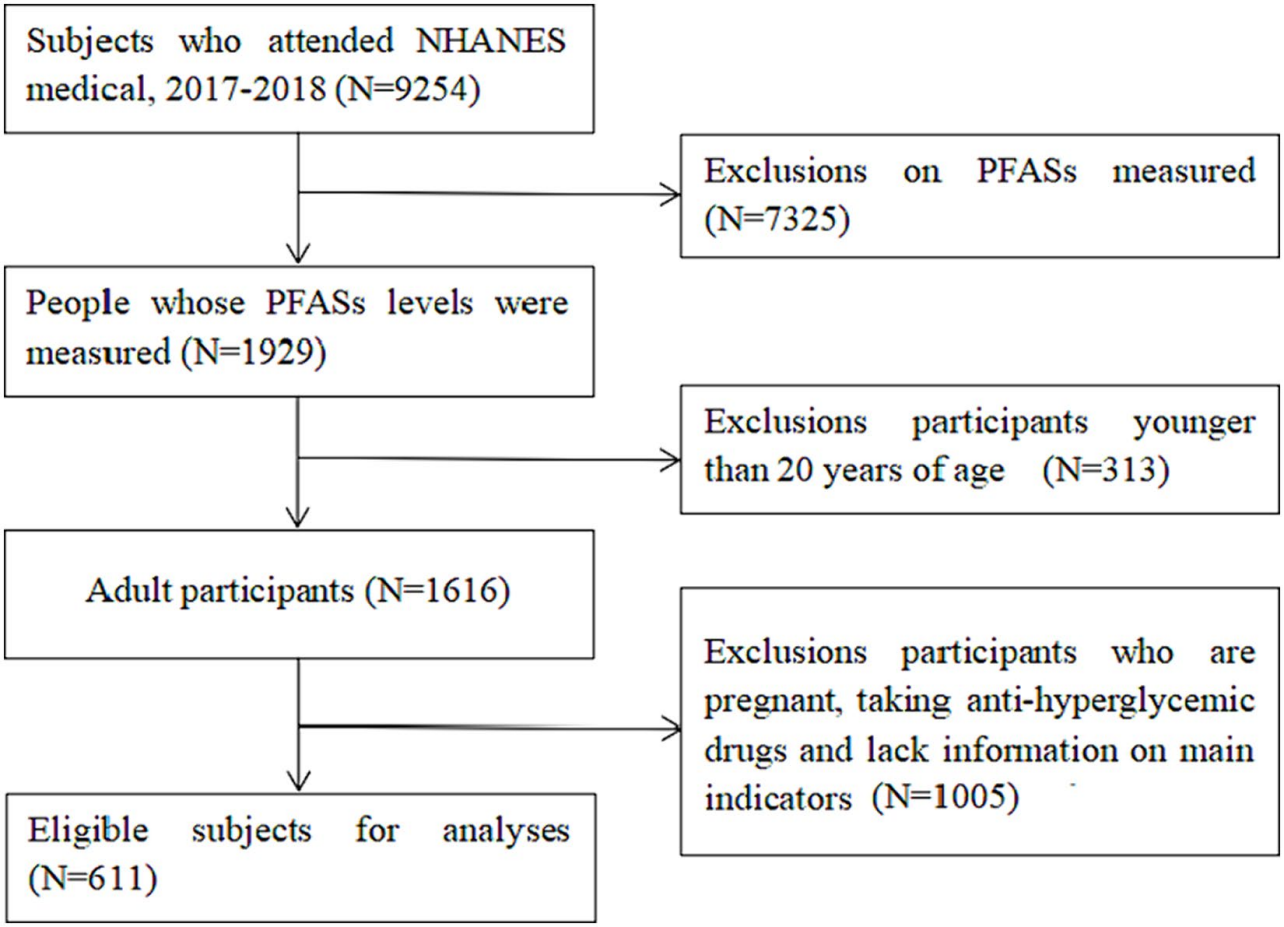
NHANES database research participants screening flow chart. (Data screening from top to bottom as indicated by the arrow).

The total sample size in 2017–2018 was 9,254, of which 1929 were tested for serum PFASs. Considering that type 1 diabetes mellitus accounts for about 90% of total diabetes in children and adolescents and is the most common form of childhood diabetes in most parts of the world (23); at the same time, pregnant women are at risk of gestational diabetes mellitus. 14% of pregnant women worldwide are affected by gestational diabetes mellitus which is a global health problem, affecting a considerable number of pregnant women (24). Therefore, 313 individuals <20 years old, 1,005 individuals who were pregnant, taking anti-hyperglycemic drugs or missed main research indices were excluded. Finally, a total of 611 individuals were included in this study. The National Center for Health Statistics Research Ethics Review Board approved NHANES, and all participants provided written informed consent. The selection process of research participants is summarized in Figure 1.

### 2.2 Covariates

The demographic database provided information on the gender (male, female), age, race (mexican-American, other Hispanic, non-Hispanic white, non-Hispanic blacks, other races), marital status (married, bereaved spouse, divorce, separation, unmarried, cohabitation), poverty, and education level (less than high school education, high School Degree, university and above). Information on weight and body mass index (BMI) were obtained from obtained from Examination database. Information on smoking, alcohol and leisure-time physical activity were obtained from Questionnaire database. Smoking status was classified as never smoking (fewer than 100 cigarettes or other tobacco products in their life), previously smoking (over 100 cigarettes or other cigarettes in their life but now quit smoking), and currently smoking (100 cigarettes or other cigarettes in their life and still smoking). Drinking status was classified as never drinking (no kind of alcohol in their life), previously smoking (drinking previously, but not in the past 12 months), and now smoking (drinking in the past 12 months). Leisure-time physical activity for each participant was categorized based on the recommended weekly amount of moderate-intensity to vigorous-intensity activity as follows: (1) below, indicating less than 150 min per week; (2) meet, indicating 150 to 300 min per week; (3) exceed, indicating more than 300 min per week.

### 2.3 Laboratory measurement methods

#### 2.3.1 Blood specimen collection

Each study participants need to meet the 8 to less than 24 h fasting criteria and draw venous blood in a fasting state. The phlebotomist collected study participant’s peripheral venous blood into 2 mL gray tubes for FPG and into 15 mL red top tubes for PFASs and insulin. Centrifuge the 2 mL gray tube to yield plasma and transfer at least 0.5 mL plasma from this tube into 2 mL vessels. Centrifuge the red top tubes to yield serum and remove serum into 5 mL sterile cryovials for PFASs and 2 mL vessels for insulin. Store under appropriate frozen (−30°C) conditions until they are tested.

#### 2.3.2 Measurement of serum PFASs concentration

Online solid phase extraction coupled to high-performance liquid chromatography-turbo ion spray ionization-tandem mass spectrometry was used for the quantitative detection of the PFASs. A total of six perfluorinated compounds, including PFOA, PFOS, perfluorodecanoic acid (PFDeA), PFHxS, PFNA, and perfluoroundecanoic acid (PFUA), were analysed in this study. Notably, the PFOA and PFOS used in this manuscript refer to the sum of linear and branched. (The description of measurements of PFASs for NHANES 2017–2018 presents n-perfluorooctanoic acid (n-PFOA), Branch perfluorooctanoic acid isomers (Sb-PFOA), n-perfluorooctane sulfonic acid (n-PFOS), Perfluoromethylheptane sulfonic acid isomers (Sm-PFOS)). The limits of detection (LOD) of the six PFASs were all 0.1 ng/mL. Following NHANES analysis guidelines, PFASs below the LOD were expressed using LOD/ $\sqrt{2}$ . Details on the analytical methodology can be found on the NHANES website^2^.

#### 2.3.3 Measurement of glucose metabolism indices

FPG were determined by hexokinase initiation using the Roche/Hitachi cobas c system (c311). Insulin was measured using the Tosoh AIA system analyzer. Homeostasis model assessment for insulin resistance (HOMA-IR) was calculated as follows: HOMA-IR = FPG (mmol/L) × insulin (mIU/L)/22.5.

### 2.4 Statistical analysis

We calculated weighted means (±standard deviation [SD]) using the NHANES primary sampling unit, strata, and weights of environmental samples for continuous variables and frequencies (proportions) for categorical variables. Means and standard deviations (SDs) for continuous variables with normally distributed distribution, medians and interquartile ranges (IQRs) for continuous variables with non-normally distributed distribution, and proportions for binary or categorical variables were displayed. The distributions of serum PFAS were generally right-skewed, therefore, were ln-transformed was conducted. Spearman correlation analysis were presented using correlation heat maps.

Cluster analysis was performed based on the concentration of PFASs. K-means algorithm is the most used clustering method, which is simple to operate, computationally efficient, so K-means algorithm was used in this study (25). First, the logarithmic transformation of PFASs concentration was performed to achieve an approximate normal distribution. After the data was standardized, the index of sum of squared error provided by Factoextra package in R 4.2.2 was used to determine the optimal number of clusters. The overall population was divided into separate subgroups using the k-means algorithm. The ratio of the average concentration in each subgroup to the average concentration in the total participants of each PFASs was calculated to further assess the exposure level. The Kruskal–Wallis H and Chi-square tests were used to compare differences in baseline information and the levels of glucose metabolism indices between subgroups. Variables showing significant differences (*p* < 0.05) were used as covariates for multiple liner regression analysis to control for potential confounding factors.

Since diabetes is often diagnosed before the age of 50 (26); people under the age of 50 are the most active workforce, the working group affected by diabetes imposes a high economic burden on the country, so the study of glucose metabolism in people under 50 is significant (27); considering that glucose metabolism indices are easily influenced by age and the age range of the study population was large, the study participants were categorized into two groups: ≤50 years old and >50 years old for correlation and regression analyses. Next, The Kruskal–Wallis H test was used to compare differences in PFASs levels between different age groups. Then, the mixed effects of PFASs were analyzed using LASSO and BKMR models.

To explore the association of single PFASs with glucose metabolism indices in the mixture of PFASs exposure, and to avoid the potential collinearity among the variables included in the regression model, the multi-PFASs exposure model was established using LASSO regression for the six PFASs based on adjusting the confounding factors of sex, age, alcohol consumption, race, leisure-time physical activity, BMI, weight and poverty ratio. The coefficient distribution of these six PFASs was used as the penalty parameter for LASSO regression path selection, and the PFASs related to glucose metabolism indices were screened using five cross-validations. Subsequently, the selected elements of PFASs were included in the multiple linear regression model and analyzed the regression coeffcients and 95% confidence intervals (95% CI). The variance inflation factor (VIF) was calculated to evaluate the multicollinearity of PFASs in the model. It is generally believed that if the VIF of an independent variable is >10, there is a multicollinearity problem between the independent variable and other independent variables.

Furthermore, we used the BKMR model as part of the sensitivity analysis and further explored the mixed effects of multiple PFASs exposure on glucose metabolism indices. BKMR utilizes a flexible non-parametric approach to assess dose–response relationships, overcoming the disadvantage that conventional methods may be limited by multicollinearity and model selection error, to more reliably assess the health effects of environmental chemical mixtures (28). This study used the BKMR model to present the cumulative effect of the mixture of the six PFASs, the univariate expose-response relationships between PFASs and glucose metabolism indices and the interactions among individual PFASs. The BKMR analysis included the same covariates as the LASSO regression and calculated the posterior inclusion probability (PIP) to quantify the relative importance of each element to glucose metabolism, with values ranging from 0 to 1. The “BKMR” and “ggplot2” packages of R4.2.2 were used to build the BKMR model and present the results. The data were combined and analyzed using R4.2.2 software, and *p* < 0.05 was presented at the significance level.

## 3 Results

### 3.1 General condition of the study participants and distribution of blood PFASs

As shown in Table 1, 611 participants were included in this study comprising 308 males and 303 females, which accounted for 50.4 and 49.6% participants, respectively. Never smokers and individuals with a history of alcohol consumption accounted for 56.3 and 91.3%, respectively, and the median age was 53 years.

**Table 1:** Basic characteristics of study participants, grouped by gender (*N* (%)/M (P25, P75)).

	Total (*N* = 611)	Male (*N* = 308)	Female (*N* = 303)	p
Age (median [IQR])	53.00 [36.00, 64.00]	54.00 [39.00, 65.00]	52.00 [34.00, 64.00]	0.200
Smoking (%)				<0.001
Now	111 (18.2)	65 (21.1)	46 (15.2)	
Previously	156 (25.5)	95 (30.8)	61 (20.1)	
Never	344 (56.3)	148 (48.1)	196 (64.7)	
Drinking (%)				0.012
Now	451 (73.8)	240 (77.9)	211 (69.6)	
Previously	107 (17.5)	51 (16.6)	56 (18.5)	
Never	53 (8.7)	17 (5.5)	36 (11.9)	
Race (%)				0.646
Mexican-American	77 (12.6)	38 (12.3)	39 (12.9)	
Other Hispanic	48 (7.9)	20 (6.5)	28 (9.2)	
Non-Hispanic White	238 (39.0)	125 (40.6)	113 (37.3)	
Non-Hispanic Blacks	143 (23.4)	75 (24.4)	68 (22.4)	
Other races	105 (17.2)	50 (16.2)	55 (18.2)	
Marriage (%)				0.113
Married	306 (50.1)	168 (54.5)	138 (45.5)	
bereaved spouse	41 (6.7)	16 (5.2)	25 (8.3)	
Divorce	81 (13.3)	38 (12.3)	43 (14.2)	
Separation	24 (3.9)	8 (2.6)	16 (5.3)	
Unmarried	104 (17.0)	54 (17.5)	50 (16.5)	
Cohabitation	55 (9.0)	24 (7.8)	31 (10.2)	
Education (%)				0.806
Less than high school education	108 (17.7)	57 (18.5)	51 (16.8)	
High school degree	140 (22.9)	68 (22.1)	72 (23.8)	
University and above	363 (59.4)	183 (59.4)	180 (59.4)	
Leisure-Time Physical Activity				<0.001
Below	365 (59.7)	159 (51.6)	206 (68.0)	
Meet	44 (7.2)	30 (9.7)	14 (4.6)	
Exceed	202 (33.1)	119 (38.6)	83 (27.4)	
BMI(median [IQR])	28.40 [24.50, 33.80]	27.80 [24.98, 31.80]	29.00 [23.95, 35.85]	0.062
Weight(median [IQR])	80.60 [67.00, 95.90]	84.50 [73.35, 98.62]	2.43 [60.10, 91.45]	<0.001
Poverty ratio (median [IQR])	2.20 [1.24, 4.25]	2.43 [1.31, 4.56]	2.03 [1.22, 4.06]	0.165
SBP (median [IQR])	125.00 [113.00, 137.00]	125.50 [116.00, 137.00]	123.00 [109.00, 136.50]	0.009
DBP (median [IQR])	73.00 [65.00, 79.00]	74.50 [67.00, 82.00]	71.00 [64.00, 77.00]	<0.001

Data are presented as the median (IQR) or frequency (percentage).

**Figure 2:**
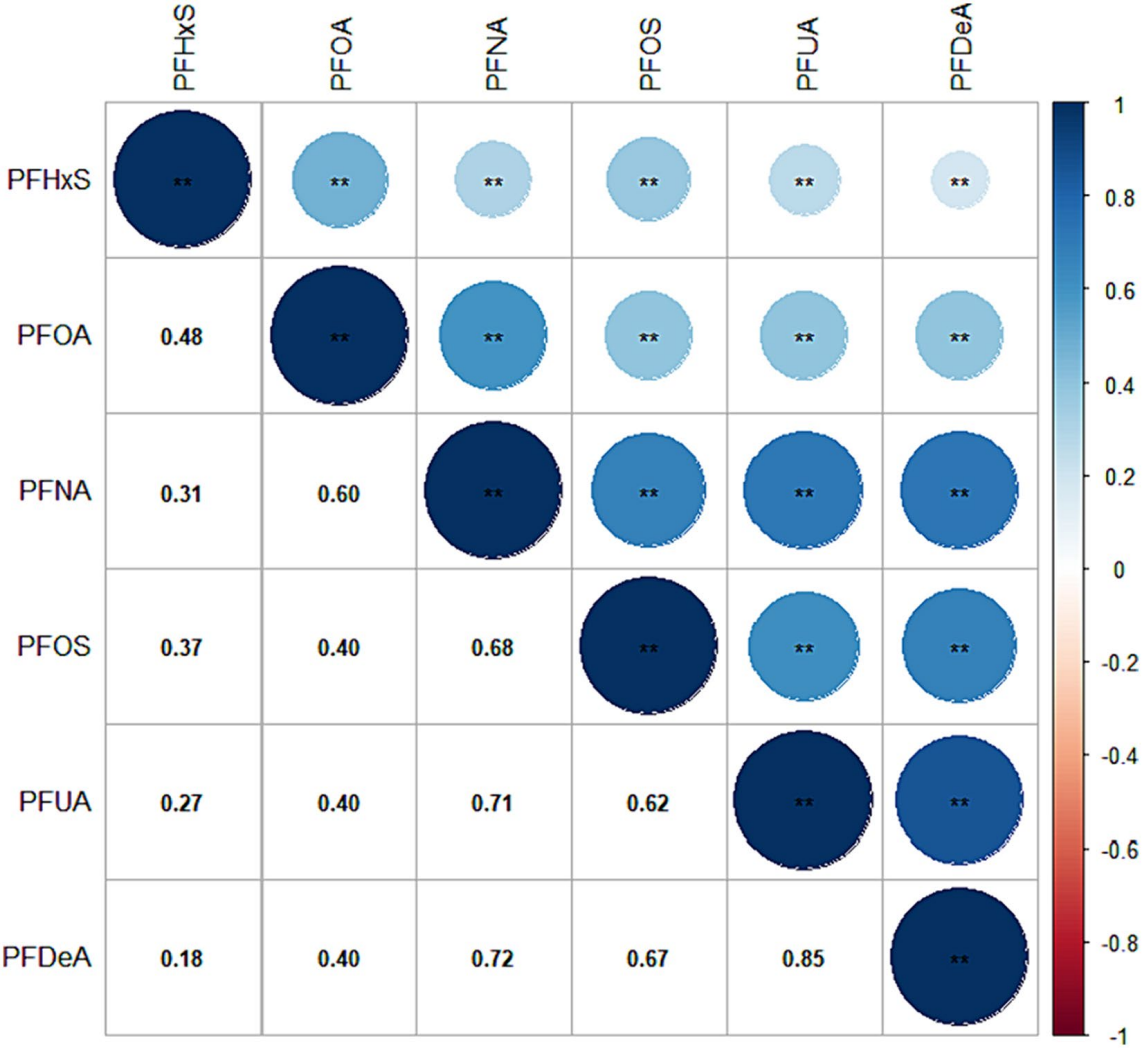
Heat map of PFAS correlation in blood. (The values in the box are the correlation coefficient, with values ranging from −1 to 1).

Except for PFUA, the blood detection rate of the other five PFASs exceeded 85%. The detection rate and concentration of PFASs in the blood of the study participants are presented in Table 2. Figure 2 illustrates that the correlation coefficients between PFDeA and PFUA, PFNA and PFUA, and PFNA and PFDeA were 0.85, 0.71, and 0.72, respectively. This suggests a strong correlation between some PFASs (*r*_s_ > 0.7).

**Table 2:** Detection rate and concentration of PFASs (ng/mL) in the population (median [IQR]).

PFASs (median [IQR])	%>LOD	Median [IQR]
PFOA	100.0%	1.47 [0.97, 2.17]
PFOS	99.7%	5.00 [2.90,8.30]
PFDeA	88.5%	0.20 [0.10, 0.30]
PFHxS	99.2%	1.20 [0.70, 2.00]
PFNA	92.5%	0.50 [0.30, 0.80]
PFUA	68.4%	0.10 [0.07, 0.20]

Data are presented as the median (IQR) or frequency (percentage).

### 3.2 Subgroup analysis of the relationship between PFASs and glucose metabolism indices

#### 3.2.1 Cluster analysis based on PFASs exposure

**Figure 3:**
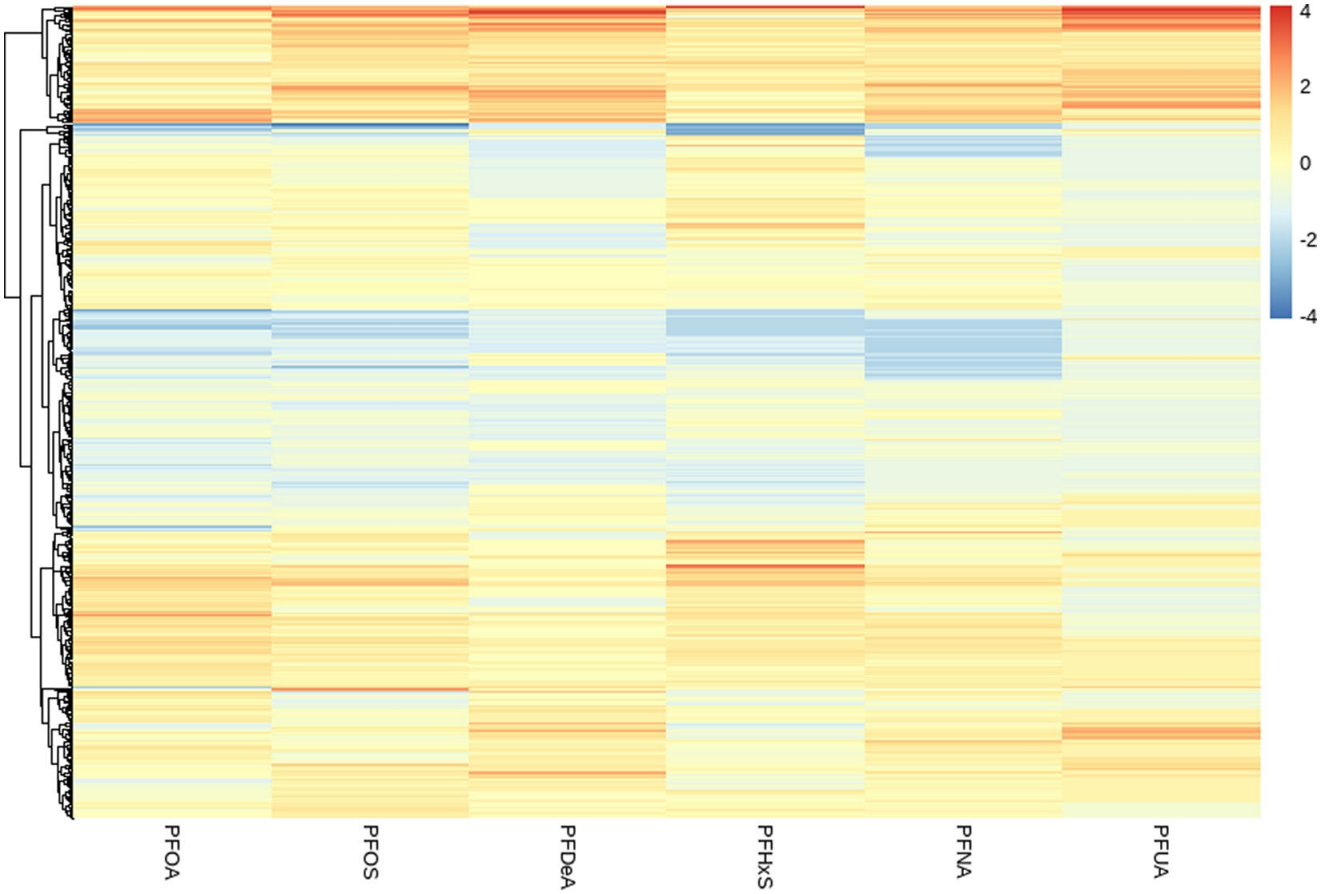
Heat map of clustering based on the concentration of PFASs in blood. (The vertical axis represents different PFASs. The horizontal axis represents the sample).

**Figure 4:**
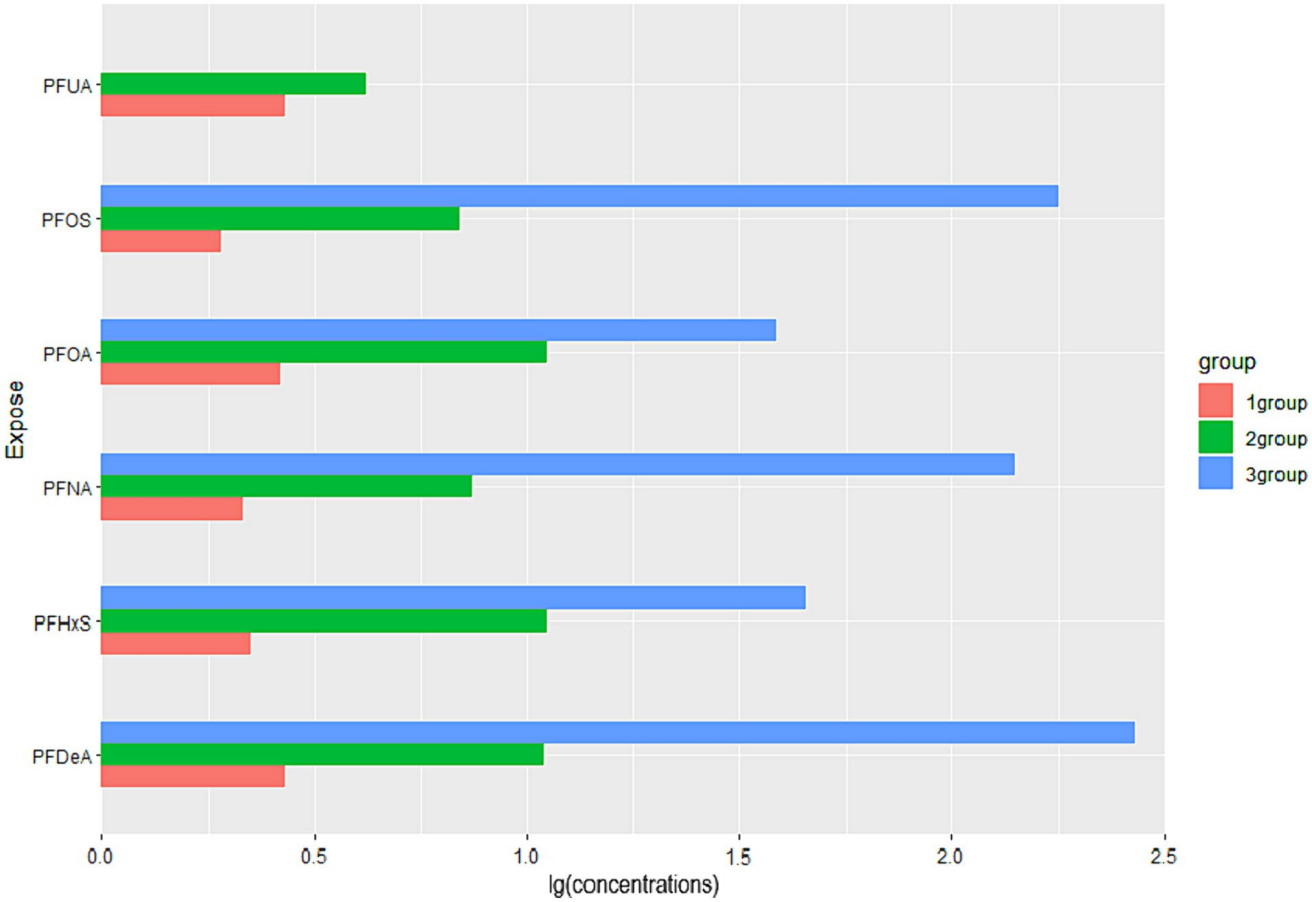
Comparison of PFASs concentrations among three exposure subgroups. (Data are expressed as the ratio of the mean concentration of PFASs in subgroups to the population mean; all groups (*n* = 6). The horizontal coordinates indicate the relative levels of the mean concentrations of the subgroup PFASs to the overall mean, and the vertical coordinates are the six PFASs).

**Figure 5:**
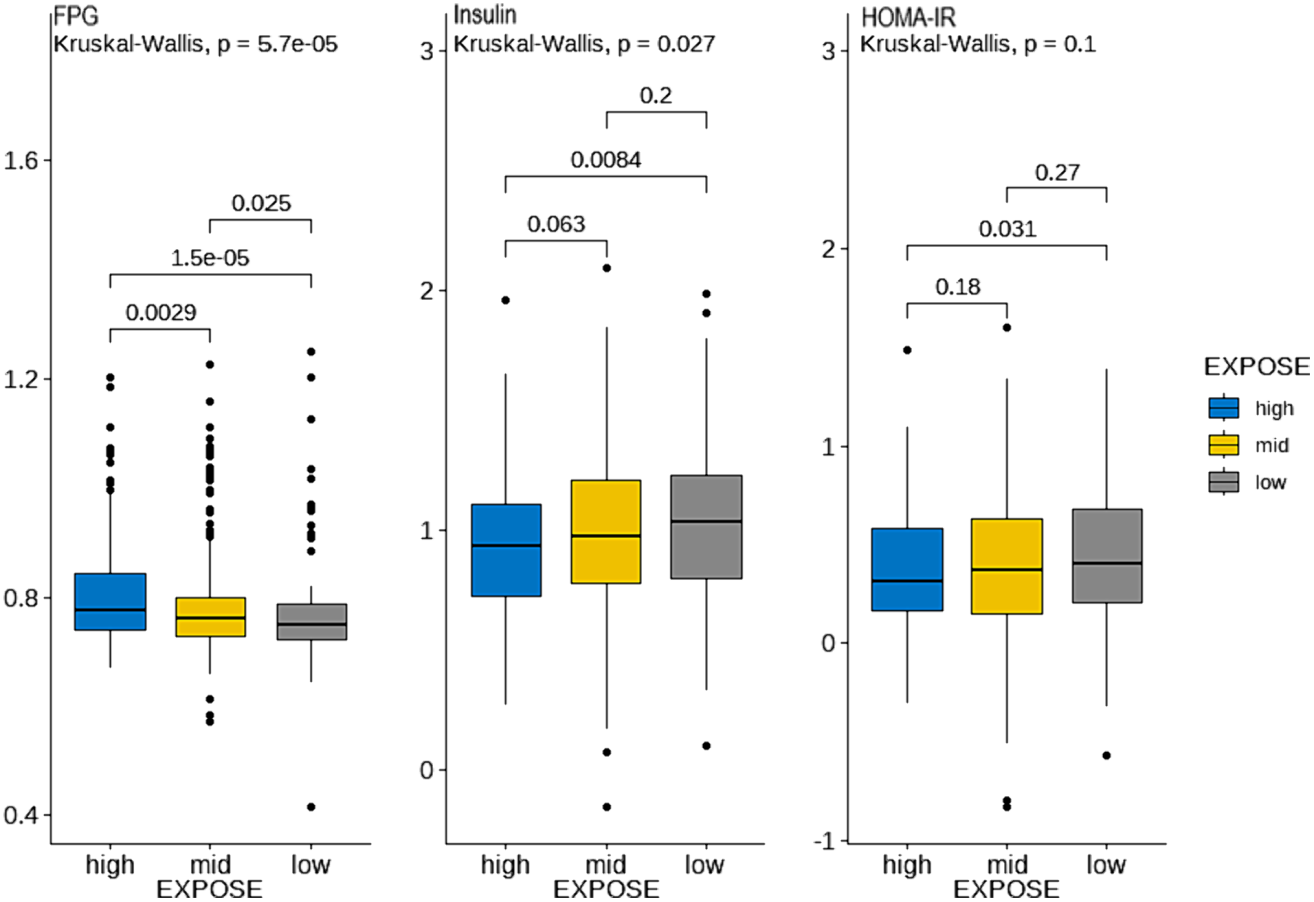
Comparison of glucose metabolism indices between the three exposure subgroups. (Blue, yellow, and gray represent the high, medium, and low exposure groups, respectively. (Data are expressed as mean ± SD; all groups (*n* = 3)). The ordinate represents the logarithmic concentration of glucose metabolism indices, and the horizontal coordinate is the exposure subgroup).

By observing the the cluster heatmap (Figure 3), we found that individuals highly exposed to one type of PFASs were also likely to be exposed to other PFASs simultaneously. As can be seen in the Figure 4, the exposure levels of the six PFASs in subgroup 3 were significantly higher than those in subgroups 2 and 1, while subgroup 2 was significantly higher than subgroup 1. From this, we identified subgroups 1, 2, and 3 as low, medium, and high exposure subgroups, respectively. Figure 5 shows the comparison results of glucose metabolism indices among the three subgroups, with statistically significant differences in FPG among all subgroups and insulin between the high-exposure and low-exposure groups. The trend of FPG and insulin levels showed statistical significance (*p* < 0.05, Table 3), indicating a linear trend between exposure and these two glucose metabolism indices. Combined with the box plots (Figure 5), it was found that FPG levels showed an elevated trend with increasing exposure to PFASs. However, insulin demonstrated a decreasing trend with increasing exposure to PFASs, indicating an association between PFASs exposure and changes in glucose metabolism levels. The weighted basic characteristics of the three subgroups determined based on cluster analysis are presented in Table 4. Statistically significant differences were observed between the groups in terms of gender, age, alcohol consumption, race, and poverty ratio.

**Table 3:** Trend test of glucose metabolism indices in three exposure subgroups.

	Linear term	Sum of squares	F	p
FPG	Unweight	0.153	17.408	<0.001
	Weighted (E)	0.147	16.682	<0.001
HOMA-IR	Unweight	0.385	3.243	0.072
	Weighted (E)	0.410	3.453	0.064
Insulin	Unweight	0.634	6.125	0.014
	Weighted (E)	0.654	6.320	0.012

Data are presented as equality of variances or equality of variances.

**Table 4:** Characteristics of the high-, medium-, and low-exposure groups (*N* (%)/M (P25, P75)).

	Low exposure group (*N* = 160)	Medium exposure group (*N* = 320)	High exposure group (*N* = 131)	p
Age (median [IQR])	41.00 [29.75, 56.00]	54.00 [38.00, 65.00]	61.00 [49.00, 73.00]	<0.001
Gender (%)				<0.001
Male	41 (25.6)	193 (60.3)	74 (56.5)	
Female	119 (74.4)	127 (39.7)	57 (43.5)	
Smoking (%)				0.216
Now	30 (18.8)	65 (20.3)	16 (12.2)	
Previously	35 (21.9)	85 (26.6)	36 (27.5)	
Never	95 (59.4)	170 (53.1)	79 (60.3)	
Drinking (%)				0.022
Now	113 (70.6)	242 (75.6)	95 (72.5)	
Previously	23 (14.4)	59 (18.4)	24 (18.3)	
Never	24 (15.0)	19 (6.0)	12 (9.1)	
Race (%)				<0.001
Mexican-American	31 (19.4)	40 (12.5)	6 (4.6)	
Other Hispanic	11 (6.9)	30 (9.4)	7 (5.3)	
Non-Hispanic White	61 (38.1)	139 (43.4)	38 (29.0)	
Non-Hispanic Blacks	35 (21.9)	69 (21.6)	39 (29.8)	
Other races	22 (13.8)	42 (13.1)	41 (31.3)	
Marriage (%)				0.100
Married	69 (43.1)	162 (50.6)	75 (57.3)	
Bereaved spouse	8 (5.0)	21 (6.6)	12 (9.2)	
Divorce	19 (11.9)	46 (14.4)	16 (12.2)	
Separation	9 (5.6)	10 (3.1)	5 (3.8)	
Unmarried	34 (21.2)	53 (16.6)	17 (13.0)	
Cohabitation	21 (13.1)	28 (8.8)	6 (4.6)	
Education (%)				0.584
Less than high school education	25 (15.6)	62 (19.4)	21 (16.0)	
High School Degree	43 (26.9)	68 (21.2)	29 (22.1)	
University and above	92 (57.5)	190 (59.4)	81 (61.8)	
Leisure-Time Physical Activity				0.031
below	94 (58.8)	177 (55.3)	94 (71.8)	
meet	11 (6.9)	26 (8.1)	7 (5.3)	
exceed	55 (34.4)	117 (36.6)	30 (22.9)	
BMI(median [IQR])	30.15 [25.30, 37.88]	28.50 [24.80, 33.50]	26.90 [23.40, 30.80]	<0.001
Weight(median [IQR])	83.35 [66.92, 102.95]	82.10 [68.97, 96.25]	73.20 [36.60, 90.50]	0.001
Poverty ratio (median [IQR])	1.69 [0.98, 2.79]	2.44 [1.35, 4.29]	3.04 [1.46, 5.00]	<0.001
SBP (median [IQR])	120.00 [109.00, 134.25]	125.00 [114.00, 137.00]	126.00 [116.00, 135.00]	0.018
DBP (median [IQR])	71.50 [65.00, 79.00]	73.00 [66.00, 79.00]	73.00 [67.00, 80.00]	0.679

Data are presented as the median (IQR) or frequency (percentage).

#### 3.2.2 Comparison of PFASs levels between the two age groups

As shown in Table 5, the differences in serum levels of six PFASs were statistically significant between the two groups with all *p* < 0.001. Except for PFUA and PFDeA, the remaining four PFASs levels were significantly higher in ≤50 year old group than in >50 year old group.

**Table 5:** Comparison of PFASs levels between the two age groups.

PFASs (median [IQR])	≤50 years old group (*N* = 278)	>50 years old group (*N* = 333)	p
PFOA	1.27 [0.77, 1.77]	1.67 [1.07, 2.47]	<0.001
PFOS	3.40 [2.12, 5.70]	6.30 [3.80, 11.20]	<0.001
PFUA	0.10 [0.07, 0.20]	0.10 [0.07, 0.20]	<0.001
PFNA	0.30 [0.20, 0.60]	0.50 [0.40, 0.90]	<0.001
PFHxS	0.95 [0.50, 1.60]	1.40 [0.90, 2.20]	<0.001
PFDeA	0.20 [0.10, 0.30]	0.20 [0.10, 0.30]	<0.001

Data are presented as the median (IQR).

### 3.3 The relationships of single PFASs and glucose metabolism indices

As shown in Table 6, among aged ≤50 years group, PFHxS (Insuliu:*β* = −0.194, *p* < 0.001; HOMA-IR: *β* = −0.132, *p* = 0.020) was found to be correlated with insulin and HOMA-IR. In the >50 years old population, PFNA exhibited a positive correlation with FPG (*β* = 0.059, *p* < 0.001); insulin and HOMA-IR were negatively correlated with PFUA (Insulin: *β* = −0.133, *p* = 0.037; HOMA-IR: *β* = −0.141, *p* = 0.041), and PFOA (Insulin: *β* = −0.159, *p* = 0.047; HOMA-IR: *β* = −0.163, *p* = 0.042) but positively associated with PFNA (Insulin: *β* = 0.191, *p* = 0.018; HOMA-IR: *β* = 0.220, *p* = 0.013). Additionally, the multiple linear regression models indicated that the VIF of all PFASs was less than 10, indicating no multicollinearity among the PFASs variables in the regression process.

**Table 6:** Regression coefficients of population glucose metabolism indices and blood PFASs concentrations (95% CI).

Age groups	Glucose metabolism indices	PFASs	β	95% CI	p	VIF^a^
≤50 years old	FPG	PFOS	0.006	(−0.019, 0.033)	0.6149	1.301
	Insulin	PFOA	−0.013	(−0.171, 0.1145)	0.874	2.238
		PFHxS	−0.194	(−0.244, −0.143)	<0.001	2.114
		PFUA	−0.028	(−0.143,0.101)	0.735	1.406
	HOMA-IR	PFOA	−0.027	(−1.934, 0.139)	0.749	2.238
		PFHxS	−0.132	(−0.243, −0.021)	0.020	2.114
		PFUA	−0.021	(−0.149, 0.108)	0.754	1.406
>50 years old	FPG	PFNA	0.059	(0.026, 0.091)	<0.001	1.112
	Insulin	PFOA	−0.159	(−0.219, −0.103)	0.047	2.613
		PFOS	0.053	(−0.080, 0.186)	0.436	3.414
		PFHxS	−0.031	(−0.160, 0.099)	0.645	2.510
		PFNA	0.191	(0.033, 0.349)	0.018	3.763
		PFUA	−0.133	(−0.258, −0.009)	0.037	2.442
	HOMA-IR	PFOA	−0.163	(−0.258, −0.092)	0.042	2.631
		PFOS	0.554	(−0.090, 0.200)	0.455	3.414
		PFHxS	−0.029	(−0.170,0.112)	0.685	2.510
		PFNA	0.220	(0.048, 0.391)	0.013	3.763
		PFUA	−0.141	(−0.277, −0.006)	0.041	2.442

a VIF refers to the variance inflation factor, which is generally accepted if the VIF of an independent variable is >10. It indicates that there is a covariance problem between that independent variable and other independent variables.

### 3.4 The effects of multiple PFASs on glucose metabolism indices

**Figure 6:**
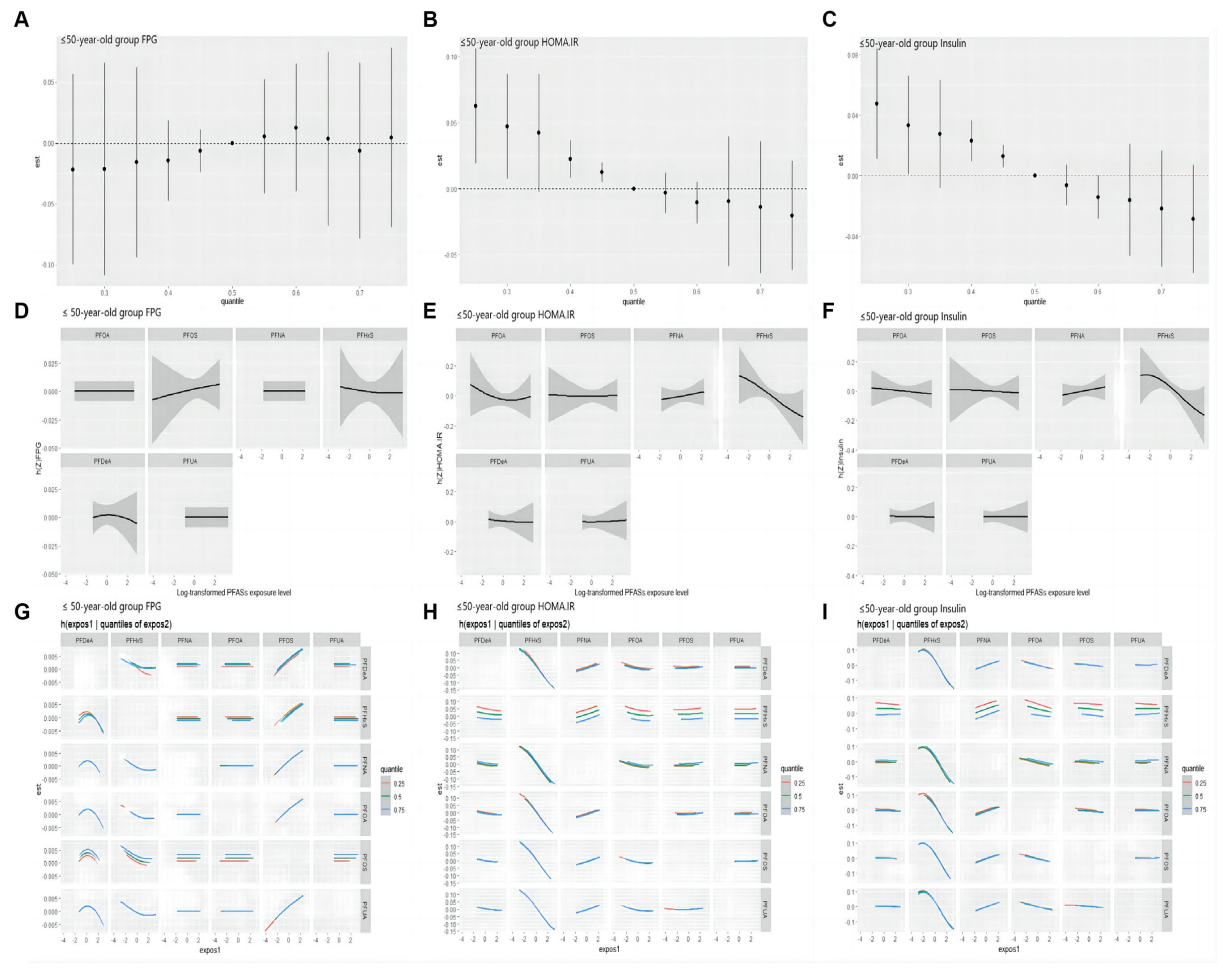
BKMR study the correlation of FPG, HOMA-IR and Insulin with PFASs in the ≤50 years-old group. **(A–C)**: overall effect (95%CI) of PFASs. *h*(*Z*) can be interpreted as the correlations of FPG, HOMA-IR and insulin with blood PFASs. **(D–F)**: exposure-response plots of FPG, HOMA-IR and Insulin against each PFAS, with other PFASs held at the median. *h*(*Z*) can be interpreted as the correlations of FPG, HOMA-IR and Insulin with blood PFASs. **(G–I)**: bivariate expose-response relationship. Each cell represented the exposure-response curve for the column PFASs when the row PFASs was fixed at 25th, 50th, or 75th percentile and the remaining PFASs were fixed at their medians.

As shown in Figure 6, in the ≤50 years-old group, the level of FPG showed an increasing trend with the increase of the total level of PFASs mixture. PFOS showed a positive expose-response relationship with FPG. Negative interaction between PFOS and PFHxS may exist. Insulin and HOMA-IR decreased with the increase of the total level of PFASs mixture. Further, PFHxS demonstrated a clear negative linear relationships with these two indices in the expose-response relationship plot, which was consistent with the LASSO regression screening results. As PFNA concentration percentiles changed from low to high, the negative effect of PFOA on insulin/HOMA-IR decreased, indicating the possibility of negative interactions between PFNA and PFOA. The results presented in Table 7 highlight the significant role of PFHxS in the relationships of PFASs on both insulin and HOMA-IR, with the highest PIPs (0.990 and 0.796, respectively).

**Table 7:** A posteriori inclusion probability (PIPs) of the effect of PFASs on glucose metabolism indices.

Variable	≤50 years-old group			>50 years-old group		
	FPG	Insulin	HOMA-IR	FPG	Insulin	HOMA-IR
PFOA	0.016	0.130	0.376	0.012	0.856	0.866
PFOS	0.488	0.096	0.374	0.048	0.576	0.586
PFDeA	0.326	0.076	0.488	0.032	0.231	0.355
PFHxS	0.136	0.990	0.796	0.016	0.688	0.458
PFNA	0.012	0.042	0.312	0.356	0.832	0.832
PFUA	0.026	0.056	0.326	0.028	0.720	0.796

Data are presented as PIP. PIP value reflects the relative importance of PFASs influence on outcome indices of glucose metabolism, and the value range is (0, 1).

**Figure 7:**
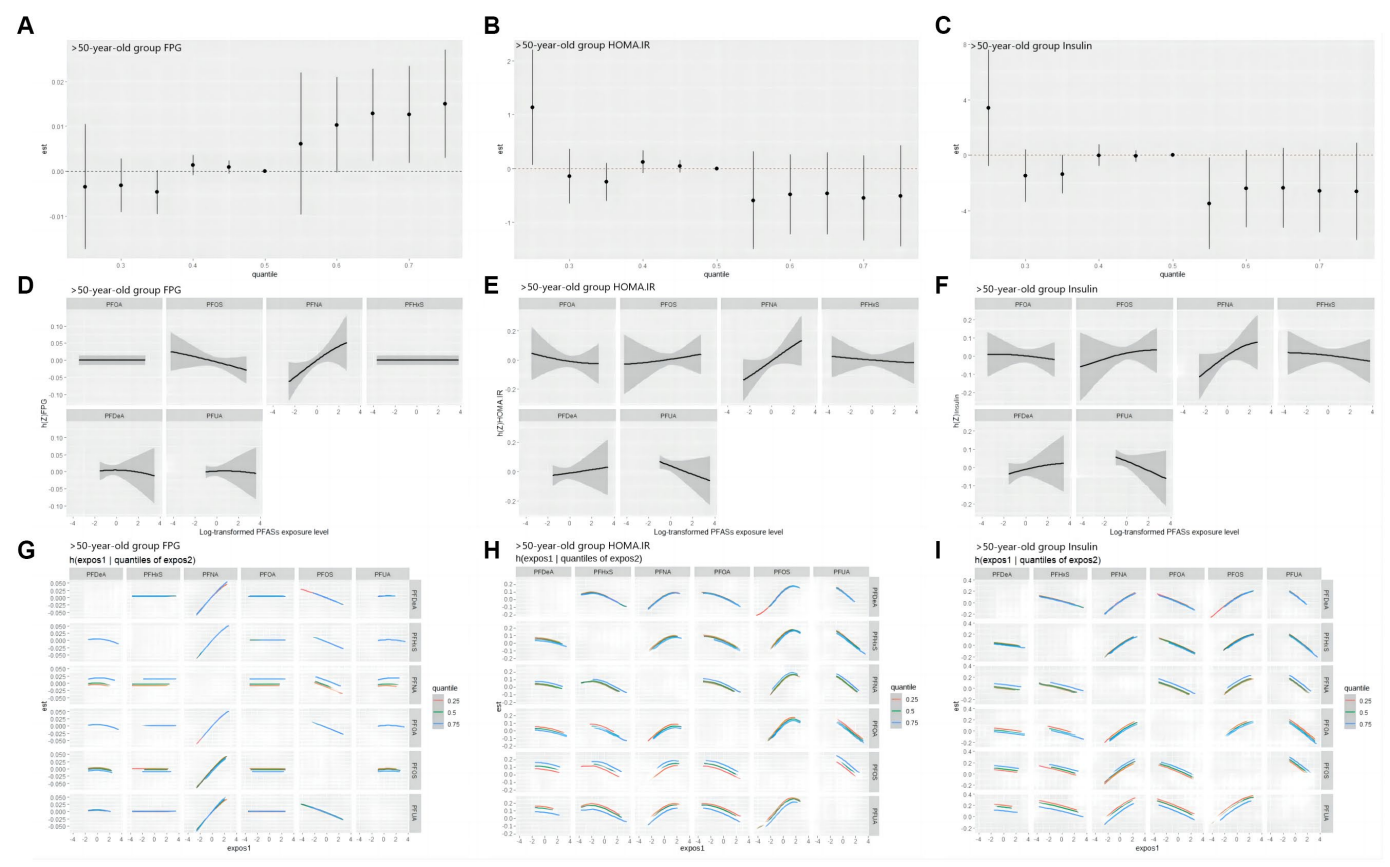
BKMR study the correlation of FPG, HOMA-IR and Insulin with PFASs in the >50 years-old group. **(A–C)**: overall effect (95%CI) of PFASs. *h*(*Z*) can be interpreted as the correlations of FPG, HOMA-IR and Insulin with blood PFASs. **(D–F)**: exposure-response plots of FPG, HOMA-IR and Insulin against each PFAS, with other PFASs held at the median. *h*(*Z*) can be interpreted as the correlations of FPG, HOMA-IR and insulin with blood PFASs. **(G–I)**: bivariate expose-response relationship. Each cell represented the exposure-response curve for the column PFASs when the row PFASs was fixed at 25th, 50th, or 75th percentile and the remaining PFASs were fixed at their medians.

As shown in Figure 7, in the >50 years-old group, the level of FPG also showed an increasing trend, corresponding to the total PFASs mixture levels. The univariate expose-response diagram showed a linear relationships between the six PFASs and the level of FPG. No interactions were observed between the PFASs. The insulin and HOMA-IR results were similar-both levels demonstrated a downward trend with the increase in the overall level of the PFASs mixture. Furthermore, the univariate expose-response relationships showed that all PFASs had linear relationship with these two indices. PFOA, PFUA and PFHxS showed a negative expose-response relationship with these two indices while PFOS and PFNA demonstrated a positive relationship, which was consistent with the LASSO regression screening results. In the bivariate exposed-response relationship plots of insulin or HOMA-IR, negative interactions were found between PFOA and PFNA/PFOS, and positive interaction was observed between PFOA and PFHxS in the bivariate exposed-response relationship plots of HONA-IR. The results presented in Table 5 highlight the significant role of PFNA in the relationships of PFASs on FPG, with the highest PIPs (0.356).

**Figure 8:**
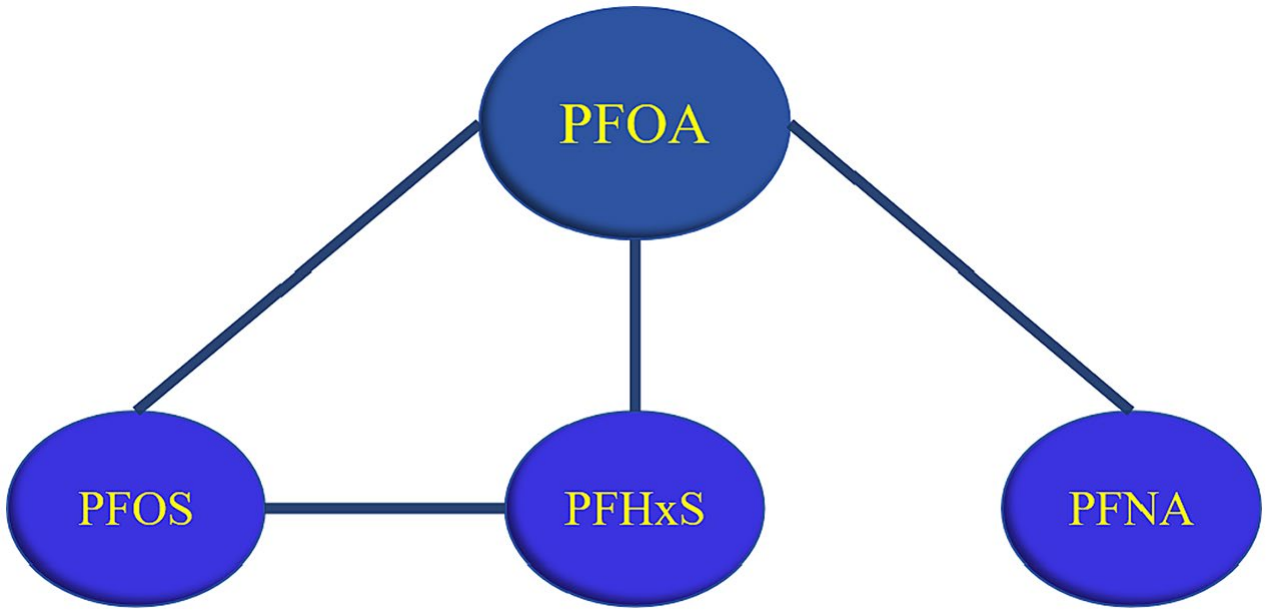
Summary of PFASs interactions. (A line between PFASs indicates possible interaction, with a black line indicating negative interaction and a red line indicating a positive interaction).

As shown in Figure 8, all possible interactions between PFASs were summarized, and it was found that PFOA could interact with multiple PFASs, and PFOA played an important role in the combined influence of multiple PFASs on glucose metabolism.

## 4 Discussion

In this study, 611 participants from the 2017–2018 NHANES cohort were selected. Cluster analysis, LASSO regression, and BKMR regression models were used to explore the relationships between the six PFASs and glucose metabolism indices. The results showed that with the increase of PFASs exposure, the FPG level showed an upward trend, while HOMA-IR/insulin demonstrated a downward trend. PFNA and FPG had a positive relationship. PFOA, PFUA, and PFHxS showed negative correlations with HOMA-IR/insulin, whereas PFNA mainly had positive correlation. Negative interactions were observed between PFOA and PFNA/PFOS, PFOS and PFHxS, while positive interactions were found between PFOA and PFHxS. Notably, PFOA can combine with various PFASs (PFOS/PFNA/PFHxS) to affect glucose metabolism indices.

PFASs exposure was closely associated with the level of FPG. The results of this study revealed that higher exposure to PFASs corresponded to a higher level of FPG, with PFNA having the greatest influence. A cross-sectional studies for adolescents and adults demonstrated that elevated serum PFNA concentration was associated with hyperglycemia (95% CI: 1.39–7.16) (29, 30). A nested case-control study also found that mixed PFASs homologs could affect glucose homeostasis by increasing 1 h glucose levels, with PFNA being the main contributor (31). The reason may be that PFNA is a kind of long-chain PFASs that is difficult to degrade and can lead to higher PPARα activation (32), which also explains the prominent position of PFNA in the relationship between PFASs and FPG.

Insulin is a protein hormone synthesized and secreted by islet β cells, which binds to target cell receptors and activates signaling pathways leading to various metabolic changes, most notably increasing glucose uptake and lowering blood glucose levels (33). Another marker of diabetes is insulin resistance that is measured using HOMA-IR. The results of this study demonstrated that exposure to mixed PFASs was associated with lower insulin and HOMA-IR levels, while PFOA, PFUA, and PFHxS were negatively correlated with both. Another study of the NHANES database also found PFASs mixture exposure were associated with decreased INS and HOMA-IR (34). Studies conducted in Cincinnati found a marginal negative correlation between PFOA levels and insulin/HOMA-IR, and another study in the New York reported a negative correlation between PFHxS and both (35, 36), which were consistent with the present study. In addition, we also found that PFNA was positively associated with both insulin and HOMA-IR levels. Zeeshan et al. analyzed data from the “Isomers of C8 Health Project in China,” and also found that PFNA was significant positive associations with insulin and HOMA-IR (37). However, there were some studies with opposite results to the present study. For example, Zeeshan et al. found significant positive correlations between PFOA, PFUA, PFHxS, and both insulin and HOMA-IR (37); some researchers found no significant correlations between PFOA/PFNA/PFHxS and HOMA-IR (2, 38). The analysis of similar and contradictory epidemiological results with this study found that different overall exposure levels in the study population may influence the association of PFASs with indicators of glucose metabolism. The results of studies analyzing populations or countries with lower concentrations of serum PFASs were more consistent with this study, concluding that there was a negative or nonsignificant correlation between PFASs exposure and insulin and HOMA-IR. For instance, the median serum concentration of PFOA was 3.8 ng/mL in NHANES (2003–2004) (2), 2.46 ng/mL in the Canadian Health Measures Survey (2007–2009) (38), and 1.47 ng/mL in this study. In studies where there were significant positive correlations, participants had a higher median PFOA concentration, such as the European Young Heart Study, which measured a median PFOA concentration of 9.7 ng/mL and 9.0 ng/mL in men and women, respectively (39). The biological mechanisms associated with PFASs and insulin resistance are unclear. Animal studies have shown that in mice exposed to PFAS, PFAS negatively regulates the protein kinase B (PKB) pathway in white adipose tissue, resulting in increased glucose and decreased insulin and insulin resistance (40). PFASs also have affinity to PPAR-γ and exposure to PFASs may also trigger expression of store free fatty acids and regulate the transcription of various insulin-related genes through activation of PPAR-γ and ultimately enhance insulin sensitivity (17, 34, 41). Although the findings from toxicology studies provide valuable insights, population data are lacking, so further research is needed to clarify the underlying mechanisms.

The results of this study showed that PFASs had different interactions in different age groups, for example, in terms of the relationships of PFASs and FPG, a negative interaction was observed between PFOS and PFHxS in ≤50 years old groups, while no interaction was found in >50 years old groups. At the same time, the differences in the serum levels of the six PFASs were all statistically significant between the two age groups in this study. Some researchers found that the combined toxic effects between PFASs may vary with the concentration (42). A study found that the toxic effect of high dose PFASs exposure experiment is not the same as that of low dose PFASs exposure experiment, that is, no effect under high dose exposure does not mean that there is no effect under low dose exposure (43, 44). Therefore, different concentrations may explain the different interaction of PFASs in different age groups. Furthermore, the results of the BKMR model indicate that there is an interaction between PFASs, especially between PFOA and multiple PFASs (PFOS/PFUA/PFHxS). PFASs are a large family containing thousands of compounds, of which PFOA is the most typical and most widely used (45). Studies show that PFOA is the final metabolite of various PFASs in the environment (46). Activation of PPAR-α is thought to play a key role in the production of toxic effects by PFASs, and PFOA is a potent agonist of PPAR-α (47). The above may be the reason for the interaction of PFOA and multiple PFASs.

Previous studies have primarily focused on the effects of a single PFAS on glucose metabolism, with limited analysis of mixed exposures, and the toxic mechanism of mixed exposure of PFASs is currently unknown. A study found that the six PFASs (PFHxS, PFOA, PFNA, PFDA, PFUA, and PFDeA) can bind to human G protein-coupled receptor 40 (GPR40), and the increase in intracellular calcium level mediated by GPR40 can promote the fusion of insulin-containing vesicles with plasma, leading to insulin secretion, disrupting glucose homeostasis and ultimately aggravating insulin resistance (48, 49). An animal study found tha the PFAS mixture could cause mitochondrial dysfunction and further disrupt glucose and lipid metabolic pathways, ultimately causing metabolic disorders (50). Further studies are needed to clarify the combined mechanism of action of PFASs in the future.

The strengths of this study are as follows: this study first used the method of cluster analysis to automatically categorize the study participants into three groups based on PFASs exposure levels. By comparing the differences in glucose metabolism levels among these exposure groups, the distribution of PFASs among the participants and the influence of PFASs exposure levels on glucose metabolism indices were effectively presented. Further, unlike previous studies on the health effects of exposure to a single PFAS, this study explores the relationship between exposure to multiple PFASs and glucose metabolism indices. This study used LASSO regression to screen PFASs variables and used the BKMR model to evaluate the overall mixed effects and interactions of multiple PFASs. These two approaches complemented each other.

However, some limitations should be recognized. First, we cannot rule out residual and unmeasured confounders (for example, total fat or high fructose dietary intake), or consumption of foods packaged with food contact materials containing PFASs, which could lead to more PFAS exposure. Additionally, the cross-sectional design could not tell the causal relationship between PFAS exposure and glucose metabolism and biological mechanisms linking PFASs exposure to glucose metabolism have yet to be established. Therefore, further experimental studies are required to explore the relevant mechanisms underlying the association of serum PFASs with glucose homeostasis and metabolic indices.

## 5 Conclusion

In this study, we analyzed 2017–2018 United States NHANES data to assess the relationships between serum concentrations of the six PFASs and glucose metabolism indices. It was found that PFOA, PFOS, PFUA, PFNA, and PFHxS could play a significant role in the relationships of PFASs and glucose metabolism. Moreover, interactions were observed between PFOS and PFHxS, and PFOA and PFOS/PFHxS/PFNA. Our study provides new evidence for the harmful effects of PFASs exposure; however, further longitudinal studies are needed to confirm these findings and clarify the underlying mechanisms.

## Data availability statement

Publicly available datasets were analyzed in this study. This data can be found at: https://www.cdc.gov/nchs/nhanes/.

## Ethics statement

The studies involving human participants were reviewed and approved by National Center for Health Statistics Research Ethics Review Board. The participants provided their written informed consent to participate in this study.

## Author contributions

QT: Writing – original draft, Visualization, Software, Methodology, Formal analysis, Conceptualization. YY: Writing – review & editing, Visualization, Software, Resources, Data curation, Conceptualization. QA: Writing – review & editing, Software, Methodology. YL: Software, Methodology, Writing – review & editing. QW: Writing – review & editing, Validation, Resources. PZ: Writing – review & editing. YuZ: Writing – review & editing. YiZ: Writing – review & editing, Funding acquisition. LM: Project administration, Writing – review & editing, Supervision, Data curation. LL: Project administration, Writing – review & editing, Supervision, Funding acquisition.

## Footnotes

^1^ https://www.cdc.gov/nchs/nhanes/

^2^ https://wwwn.cdc.gov/Nchs/Nhanes/2017-2018/PFAS_J.htm
